# Development of hybrid composite by integrating functionalized multi-walled carbon nanotubes (*f-MWCNTs*) with glass fiber reinforced polyester composite

**DOI:** 10.1371/journal.pone.0279647

**Published:** 2022-12-29

**Authors:** Shaban Gul, Muhammad Abdullah, Mahnoor Zafar, Irshad Ali, Nehar Ullah Khan, Mohammad Younas, Mashallah Rezakazemi

**Affiliations:** 1 Department of Chemical Engineering, Faculty of Mechanical, Chemical and Industrial Engineering, University of Engineering & Technology, Peshawar, Pakistan; 2 CAS Key Laboratory of Urban Pollutant Conversion, Institute of Urban Environment, Chinese Academy of Sciences, Xiamen, China; 3 Faculty of Chemical and Materials Engineering, Shahrood University of Technology, Shahrood, Iran; National Textile University, PAKISTAN

## Abstract

The current work presents the development of hybrid glass fiber reinforced polyester resin (GFRPs) composite. The composite integrates functionalized carbon nanotubes (*f-MWCNTs*) with glass fiber (GF) using polyester resin as a media. Hand lay-up method was adopted to prepare GFRPs samples in the form of rectangular sheets. Morphological characteristics of the GFRPs were investigated through scanning electron microscopy (SEM), to analyze the *f-MWCNTs* distribution and agglomeration of the developed composite’s surface due to varying concentrations from 0.0 to 0.5 wt.%. Fourier transform infrared spectroscopy (FTIR) and X-ray diffraction (XRD) were performed to confirm the presence of *f-MWCNTs* in the developed GFRPs. Sample with 0.4 wt. % *f-MWCNTs* showed the highest tensile strength and impact energy of 79 MPa, indicating a 31.66% improvement and 1.6 Nm with 77% improvement, respectively as compared to the control sample (0.wt.% *f-MWCNT*). The same sample also showed the thermal stability till 390 °C as measured through thermogravimetric analysis (TGA). Deposition of extra 10 layers initially increased the composite strength from 40 MPa to 128 MPa, however further increase in layers to 15 resulted decrease in strength to 100 MPa due to the poor interaction between the polyester resin and GF. The addition of *f-MWCNTs* in the composite effectively strengthens the interfacial bonding, which significantly improved the tensile and impact strength of the composite, making it tougher and thermally stable. However, further increase in the concentration of *f-MWCNTs* degraded the mechanical properties of developed composite such as compressive strength because of agglomeration of these nanoparticles and void formation in the composite.

## 1. Introduction

Composites emerged as distinct materials, purposefully designed and constructed polyphase materials such as fiber-reinforced polymers [1]. High-performance fiber-containing composites are widely utilized in various industrial applications like transport, construction and chemical process industries [2]. These fibrous materials include glass and carbon fibers, contained in a compliant polymeric resin such as epoxy and unsaturated polyester resins [3, 4]. Light in weight with high specific stiffness and strength, dimensional stability, superior electrical characteristics, and excellent corrosion resistance are essential structural characteristics of these composites, making them distinct from other materials [5, 6].

As part of the resin-based composites, polyester resins are easy to handle, low in cost, dimensionally stable, and possess outstanding mechanical, chemical, and electrical properties, making them suitable materials for the development of cost-effective fiber-reinforced polymeric composites [7, 8]. Every year, due to its various types of industrial utilization, several billion tons of polyesters are being produced [4]. It serves as the principal resin matrix in sheet molding compounds (SMC) and bulk molding compounds (BMC) [7, 9]. Due to low viscosity, polyester is also used as a filler for the reinforcement of various composites to lower the cost of the final composites by 50% [10]. Because of the lightweight structures, faster drying time and high-performance chemical resistivity appropriate stability, and excellent corrosion resistance of the resin-based polymeric composites, these composites show appealing potential for their use in various applications [5–7]. However, due to the very low thermal stability, structure deformation, and limited scale strength of the polymeric composites, their employment is limited for a broader range of conventional and advanced materials applications [11]. To further improve the shortcomings of these resin-based composites, it is necessary to develop either way the products by combining them with other binary materials that can strengthen their properties or use high-cost materials in combination. Introducing some binary functional groups or elements into the main chain of the resin-based product is an effective way to improve the performance of polymeric composites [12]. One way in search to develop high strength polymeric composites is to mix polymer resin with reinforcement material like glass fiber, however, it alone cannot fulfill the requirement. Instead, adding a third component as a nanofiller will rectify the shortcomings in developing suitable polymeric composite [13]. Thus, such polymeric composites with higher mechanical properties can be made by incorporating glass fiber with filler materials such as carbon nanotubes (CNTs) into the polyester resin [14]. Due to their low cost and easy availability, glass fibers are currently the most widely utilized reinforcement materials for various thermosetting composites [15]. Along with the use of glass fibers, the second binary material, which is under consideration is CNTs which acts as a good candidate for nano-reinforcing materials [16–19]. Moreover, nanoparticles like silica, silver and carbon black were utilized in polyester resins to improve different properties like, corrosion resistivity, and nano particles like montmorillonite nano clays were utilized for higher thermal stability and mechanical strength [4].

CNTs are distinguished by their mechanical, electrical, magnetic, optical, and thermal properties, having elastic moduli and strength ranging from 0.3–1.0 TPa and 10–500 GPa, respectively [8, 20, 21]. Due to these properties, in addition to the high aspect ratio and low density, CNTs are considered and utilized as suitable nano-reinforcing materials in connection with other materials for composite formation [8, 16, 17]. However, due to inadequate interfacial interaction, and Van der Waals interaction between CNTs chains, the full potential of CNTs as reinforcement material has been severely hampered [22, 23]. CNTs dispersion differs from other conventional fillers and exhibits nanoscale diameter and high aspect ratio (>1000) [23–25]. Therefore, it is necessary to modify the surface properties of CNTs, preferably through functionalization where CNTs are treated with a strong acid such as nitric acid (NHO_3_) or sulfuric acid (H_2_SO_4_) or a mixture of both [26, 27]. The carbon-carbon bonded network of the graphitic layers in CNTs is broken during this treatment, known as oxidation, allowing the introduction of oxygen units in the form of carboxyl, phenolic, and lactone groups [28]. Various researchers have performed several studies on the mechanical performance of CNT-modified composites. For example, Fan *et al*. added multiwall carbon nanotubes (MWCNTs) with glass fiber-reinforced epoxy composites and found that the interlaminar shear strength (ILSS) of composites increased [29]. Seyhan *et al*., achieved 8 and 11% increases in fracture toughness and ILSS, respectively, with 0.1 wt% MWCNTs on*e*-glass non-crimp fabric-modified polymer composites [30]. The reported results show that the addition of MWCNTs as a third-phase reinforcement material positively affects the mechanical performance of composites. However, further research is desired to investigate the agglomeration of nanoparticles and void formation in the final product and to find the optimal concentration and submission layers of nano reinforcement in the polymer matrix [26]. Several other researchers also studied the mechanical enhancement of CNT-polymer composite [19, 31–34]. However, their results vary due to different materials, CNTs concentrations, and varying processing conditions.

Functionalization of the MWCNTs is also one of the techniques to significantly increase the solubilization of the carbon nanotubes give rise to better homogeneous dispersion in various material-related applications [25]. Functionalization decreases the van der Walls forces of attraction thus facilitates the solubility of the CNTs, thus making functionalized MWCNTs *(f-MWCNTs)* capable of manipulation and processing for better dispersion, especially in polymeric composite makings [35, 36]. Moreover, different functional groups attached to the CNTs results in different properties. Hydroxyl-functionalized MWCNTs are more effective for homogeneous dispersion in polyester resin and contribute to better mechanical properties in final product as compared to carboxyl group-functionalized MWCNTs [25].

The functionalization of CNTs is also aimed to increase its interaction with polyester resin to avoid the agglomeration of CNTs and prevent the formation of voids in the final product [37]. The mechanical performance of CNT-modified polymer composites has been studied by various researchers [23, 24]. However, to the best of the authors knowledge, the effect of these functionalized nanoparticles addition in glass fiber reinforced polyester resin composite has not been studied so far. Literature also shows that, CNTs rather than their functionalized forms have been utilized [14, 15, 36]. In this work, polyester, polyester resin, was utilized as a media in making GFRPs modified with *f-MWCNTs* due to its light weight, ease of operation, low cost and easy availability.

In this current work, the functionalization of the MWCNTs has been done using the acid functionalization method. Functionalized MWCNTs were integrated with glass fiber reinforced polyester composite. The study compared the mechanical properties of glass fiber, CNTs, and polyester with glass fiber and polyester. A detailed analysis of the effect of functionalization with the mechanical property and surface morphology is presented. A fiberglass polymeric composite was developed with enhanced mechanical properties using a cheap and affordable polymer with a lower and lighter concentration of *f-MWCNTs*. Hence the effect of *f-MWCNTs* addition in polyester resin has not been studied so far and either epoxy or a combination of both epoxy and resins have been investigated. Literature also shows that CNTs rather than their functionalized forms have been utilized in most cases. In the search to find a durable and easily affordable polymer amongst all previously researched media, polyester resin (polymer) was selected. It is quite lighter in weight and is cheaper, making it easily affordable [14, 15, 36]. The *f-MWCNTs* properties to act as a micro filler to improve the polymer mechanical properties in a composite greatly dependent on the amount of CNTs used which were figured out as a high amount of *f-MWCNTs* would make the composite fragile and flexible.

## 2. Materials and methods

### 2.1 Chemicals and reagents

Unsaturated polyester resin (Fiber Glass Development Corporation), methyl ethyl ketone peroxide (MEKP) (Fiber Glass Development Corporation), non-woven glass fibers (Taishan Fiberglass Inc., E-type, surfaces 300 g/m^2^), MWCNTs (average diameter of 10 to 20 nm), absolute ethanol (Merck), nitric acid (63%, Sigma Aldrich), sulphuric acid (98%, Sigma Aldrich) and cobalt were used. All chemicals were of analytical grade. Aqueous solutions were prepared using ultra-pure deionized water of resistivity 18.2 MΩcm.

### 2.2 Acid functionalization of carbon nanotubes

**Fig 1:**
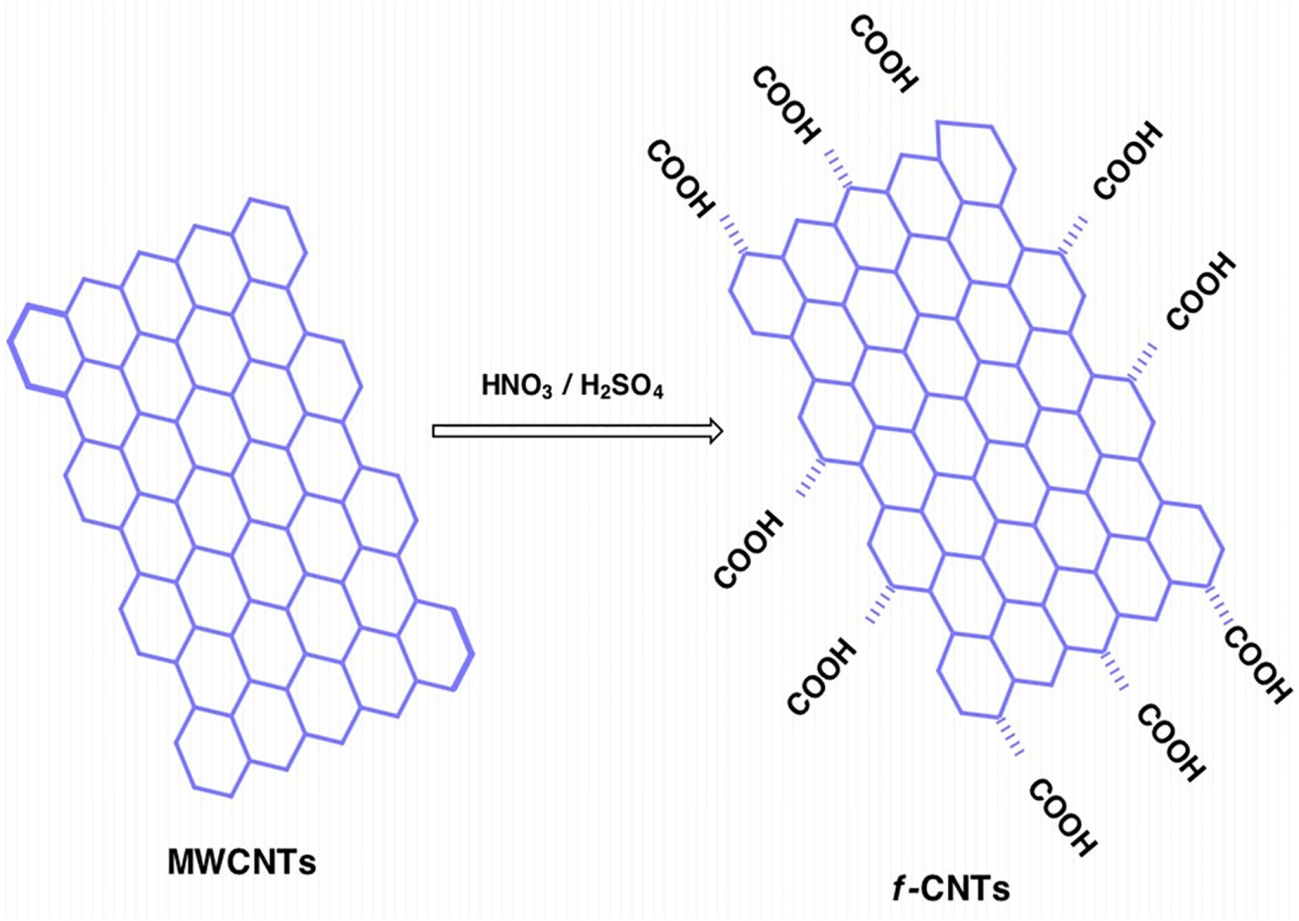
Functionalization reaction of the MWCNTs.

The solution of *f-MWCNTs* was prepared using the covalent acid functionalization method [35, 36]. 3 g of CNTs were treated with 150 ml solution of a mixture of nitric acid and sulfuric acid in a 1:3 ratio at 140 °C for 2 h to attach oxygen functional groups to the carbon surface in the form of carboxylic acid [33]. The attachment of oxygen functionalization groups is shown in Fig 1. After the acidic treatment, the mixture was allowed to cool for some time. Then, the mixture was filtrated and washed with hot distilled water to remove the acid solution until the PH level of the CNTs reached 7. These acid-modified CNTs are termed as *f-MWCNTs*. The *f-MWCNTs* were dried at 100 °C for 24 h to remove moisture. After drying, 100 ml solution of 60 wt. % *f-MWCNTs* in ethanol was prepared and sonicated for 1 h to obtain a homogenous solution of *f-MWCNTs* in ethanol [33, 38].

### 2.3 Sample preparation

**Fig 2:**
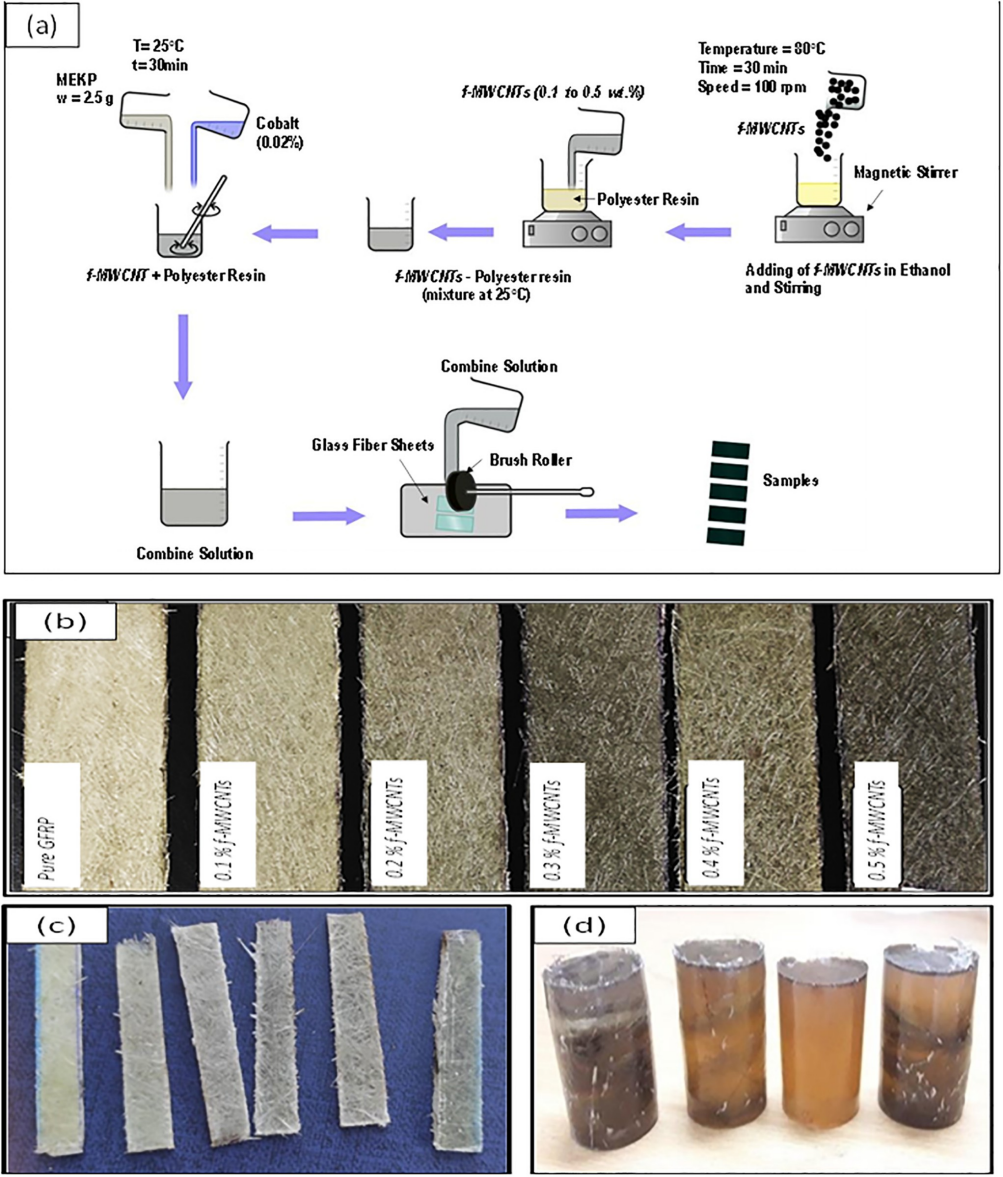
(a) Schematic of the sample preparation, (b) developed GFRPs samples modified, (c) samples for impact strength tests, (d) samples for compressive strength tests.

For sample preparation, hand lay-up method was used [39]. Five solutions of *f-MWCNTs* and polyester resin were prepared with different concentrations of *f-MWCNTs* (0.0 to 0.5 wt. %). Beaker was placed on a magnetic stirrer at 100 rpm and 80 °C for 30 min for proper dispersion of *f-MWCNTs* in polyester resin and evaporation of ethanol followed by cooling at room temperature (Fig 2a) [25]. Then, MEKP as a curing agent was added in the ratio of 1.25 g MEKP/100 g resin along with 0.02 wt.% cobalt as an accelerator and stirring was appropriately done [4, 25]. Glass fiber sheets were cut in the required dimensions. Glass fiber sheets was placed in an open mold having non sticky surface and then saturated with a wet polyester resin containing *f-MMCNTs*. The prepared solution was employed on the surface of the sheets by brush. For proper curing, these sheets were kept for 4–5 h. Five samples of dimensions 270×90×2.5 mm^3^ were prepared, with different wt % concentration of *f-MWCNTs* from 0.0 to 0.5 wt. % (Fig 2b). Moreover, the same method was used in making samples for the compressive strength test while the concentration of *f-MWCNTs* was kept constant and layers of glass fiber sheets were varied (Fig 2d). The samples were made in a cylindrical mold. The weight percentages of the components are shown in Table 1. Furthermore, these samples were cut in different sizes according to their ASTM standards for different tests. Tensile tests were conducted according to ASTM D3039 with sample dimension of 250×20×2.5 mm^3^. Charpy Impact test is done according to ASTM D-256 on the Charpy impact tester with a sample dimension of 48×8×2.6 mm^3^ (Fig 2c). Compressive strength test for multilayer deposition was performed according to ASTM D695 with cylindrical sample dimensions of 12.7 mm in diameter and 25.4 mm long with *f-MWCNTs* from 0.0 to 0.5 wt. %.

**Table 1:** Sample designation and weight percentages of glass fiber, polyester, and *f-MWCNTs* in the hybrid samples.

Samples	wt. % f-MWCNTs	wt. % glass fiber sheets	wt. % polyester resin	wt.% Hardener MEKP	wt.% Cobalt accelerator
**1**	**0.0**	**15**	**83.7**	**1.25**	**0.02**
**2**	**0.1**	**15**	**83.6**	**1.25**	**0.02**
**3**	**0.2**	**15**	**83.5**	**1.25**	**0.02**
**4**	**0.3**	**15**	**83.4**	**1.25**	**0.02**
**5**	**0.4**	**15**	**83.3**	**1.25**	**0.02**
**6**	**0.5**	**15**	**83.2**	**1.25**	**0.02**

### 2.4 Characterization and analysis

**Fig 3:**
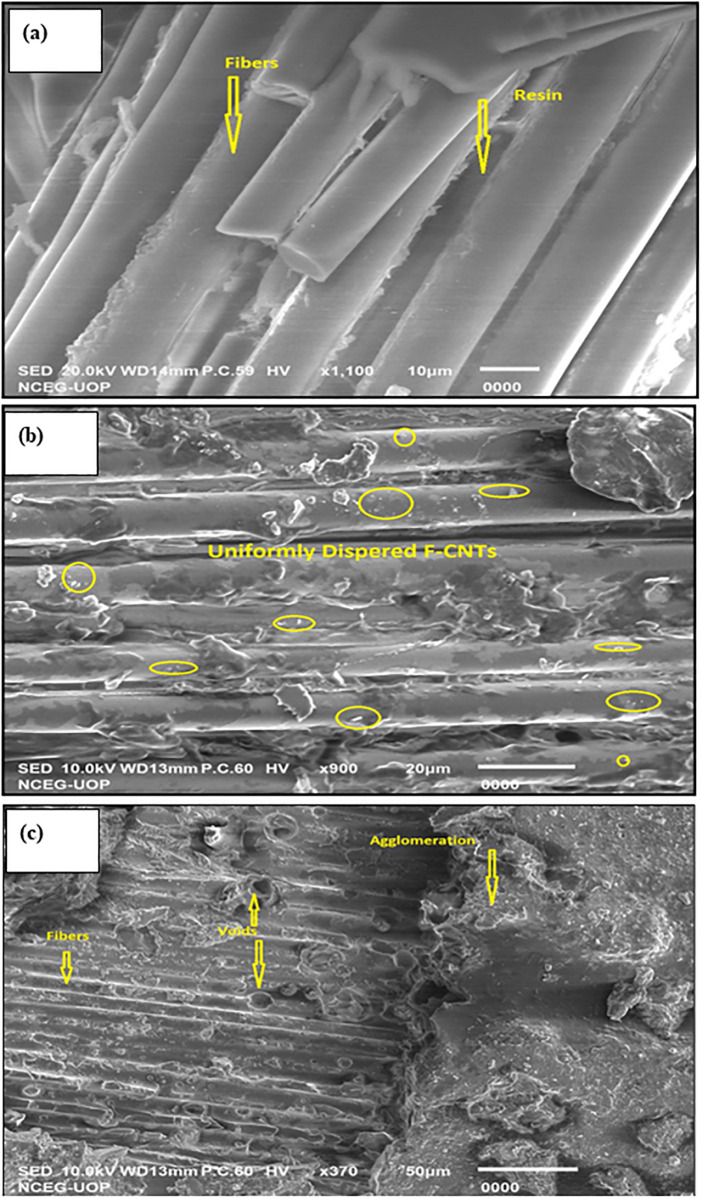
SEM images of the samples (a) virgin sample (b) 0.4 wt. % *f-MWCNTs* (c) 0.5 wt. % *f—MWCNTs*.

The surface topography/morphology and spread of *f-MWCNTs* in the developed composite were investigated through scanning electron microscopy (SEM) using a “JEOL, JSM-IT 100, Japan model SEM”. This surface characterization and morphology have further been explained in the results and discussion section with the help of Fig 3. Fourier transform infrared spectroscopy (FTIR), and X-ray diffraction (XRD) were performed to investigate the possible attached functionalized groups of MWCNTs in the final product. The XRD analysis of the developed composite’s surface (control without *f-MWCNT* and with *f-MWCNT*) was carried out using JDX-3532 model X-ray diffractometer, JEOL Japan, at CuKa (wavelength = 1.5418 Å) radiation. Infrared spectra were investigated by an IR Prestige-21 FTIR Spectrometer in the range of 500–4000 cm^−1^. XRD and FTIR were only done for 0.4 wt.% *f-MWCNTs* composite samples because their mechanical properties and stability were higher than other tested samples. The mechanical properties of the samples were also investigated by measuring their tensile and compressive strength by performing tensile and impact energy tests. Thermal stability was measured through thermogravimetric analysis (TGA).

## 3. Results and discussion

### 3.1 Surface characterization

SEM investigation was performed for the surface morphology i.e., the structure of the control and *f-MWCNTs* modified GFRPs to examine the role of *f-MWCNTs* in modifying their mechanical properties.

The distribution of CNTs on the sample surface is vital in enhancing its mechanical properties. The extent of reinforcement provided by the *f-MWCNTs* is limited due to specific reasons, such as the agglomeration of *f-MWCNTs* and the presence of voids in composites [25]. The factors which play an important role are *f-MWCNTs* aspect ratio, alignment, dispersion, and interfacial interaction, so it is essential to study the morphology of *f-MWCNTs* in composite GFRPs [26, 27]. SEM can get the surface structure and morphology of samples up to 0.1 nm resolution which is very helpful in getting insight into the developed samples [28]. To prevent the agglomeration, CNTs were functionalized to decrease the attraction among CNTs and increase their interaction with the polyester media. Furthermore, high-speed magnetic stirring was applied to the mixture of polyester and *f-MWCNTs* to get a uniform matrix. However, as the concentration of *f-MWCNTs* in polyester was increased the attractive forces among these nanoparticles overcome the effect of functionalization and caused the agglomeration in sample. Such agglomeration can be observed in Fig 3.

The surface distribution of *f-MWCNTs* and void formation was investigated for different samples. Fig 3 shows the surface morphology of the prepared composite samples i.e. with (a) virgin sample (no *f-MWCNTs)* (b) and *f-MWCNTs* with 0.4 wt. % (c) and 0.5 wt. %. It is clear from the SEM images that fibers are smoothly and uniformly dispersed in the resin matrix.

SEM image of 0.4 wt. % Fig 3b shows a uniform distribution and homogeneity of *f-MWCNTs* in the resin matrix and on the fiber surface. There is good interaction between the nanotubes, polyester resin, and fibers. In addition, no voids were observed. Therefore, it has increased tensile strength. However, a further increase in *f-MWCNTs* concentration results in decreased tensile strength due to the presence of voids and agglomeration of the *f-MWCNTs*, as shown in Fig 3c. As the concentration of nanoparticles increases, its interaction with the polyester media decreases. These voids and agglomeration cause improper interaction among the nanoparticles, resin, and fibers, ultimately decreasing the composite’s strength. Void formation resulted from the non-presence of MWCNTs element near that zone which has weakened the surface and resulted in lower strength, as reported in the literature [40].

### 3.2 FT-IR analysis

**Fig 4:**
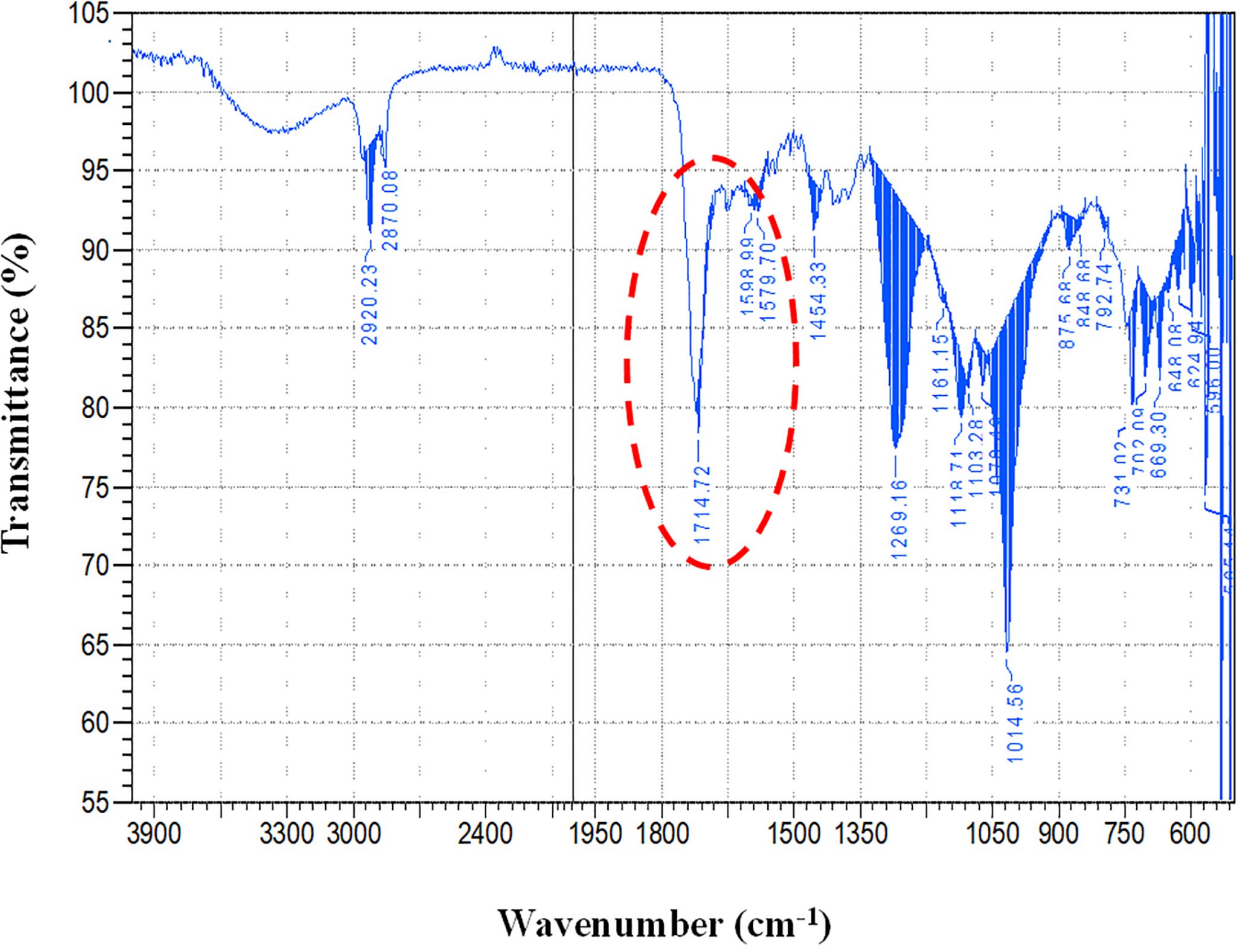
FTIR analysis of the *f-MWCNTs* modified GFRPs composite containing 0.4 wt. % *f-MWCNTs*.

FTIR spectra obtained from samples provide insight into the level of surface modification. Functional groups such as carboxyl and carbonyl on the MWCNT surface give different spectral peaks. FTIR analysis of the *f-MWCNTs* modified GFRPs was done to confirm the presence of *f-MWCNTs* in the modified composite surface. The spectrum was recorded on an FT-IR (Thermo Nicolet Avatar 320 spectrophotometer USA) using the 600 to 4000 cm^-1^ range ATR technique. The peak observed at 1579 cm^-1^ (indicated by the circle) confirms the presence of *f-MWCNTs* in composite [41]. Bands shown in Fig 4 at 2922 and 2900 cm^-1^ represent benzene rings.

The polyester peak is supposed to be around 917 cm^-1,^ but the disappearance of the band in the spectrum shows an infusion of *f-MWCNTs* into the resin [42]. In another study, it has been shown that the pristine MWNT exhibits the peaks of C-C bond stretching appeared in the range of 3000–2800 cm^-1^, but after electron-beam irradiation, more than 1000 kGy showed new peaks at 1782 cm^-1^ due to the C = O bond resulting from C = O stretch of the carboxyl and carbonyl groups [8, 43].

### 3.3 X-ray diffraction

**Fig 5:**
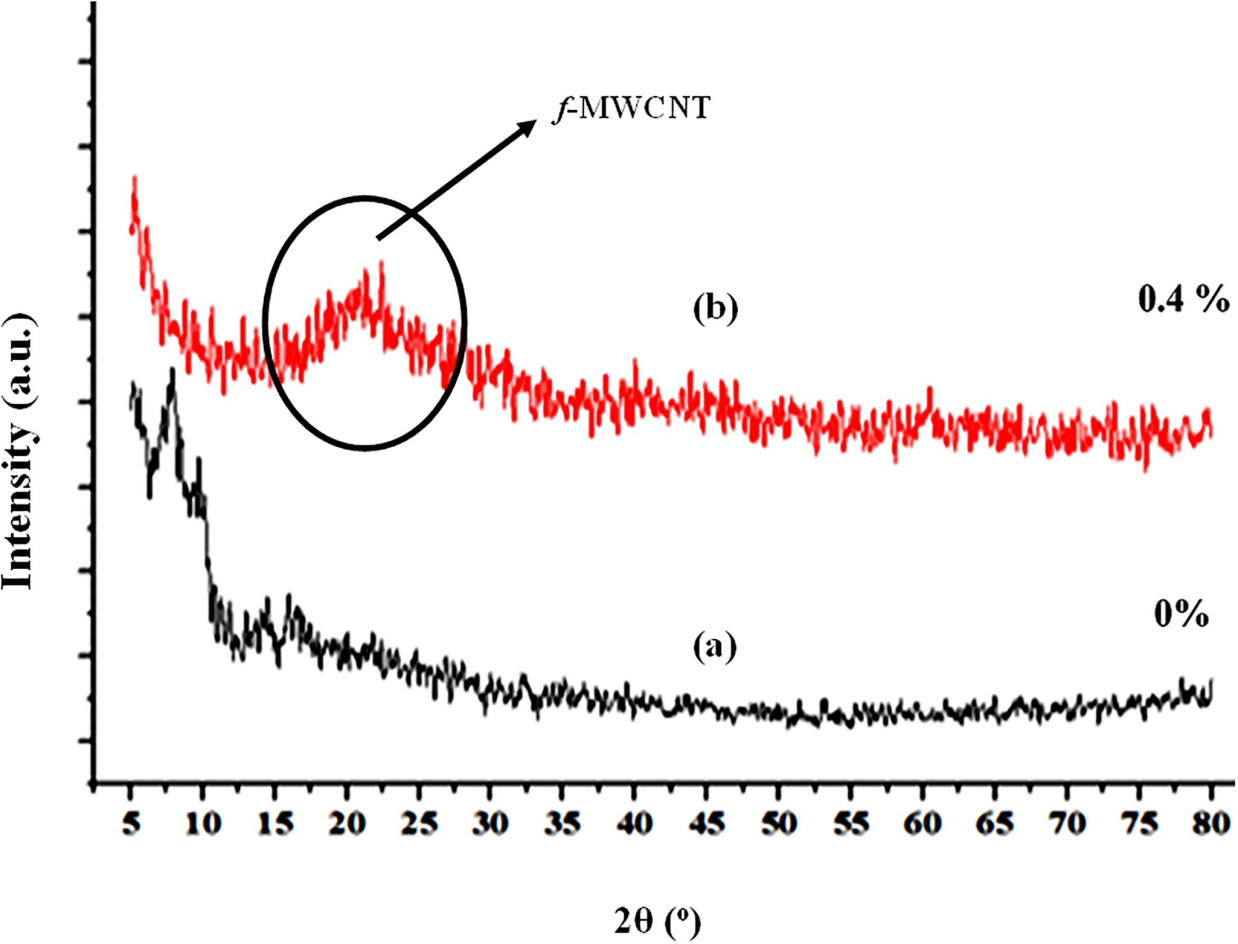
XRD pattern of (a) virgin sample (b) 0.4 wt. % *f-MWCNTs* glass fiber composite.

The polymer composite’s strength, modulus, and shrinkage properties are determined by the distribution in orientation and alignment of the *f*-*MWCNT* and the polymer matrix. X-ray Diffraction (XRD) is an appropriate tool for determining this parameter [44]. XRD of the developed composite samples was performed to investigate the surface structure of deposited *f-MWCNTs* on the composite surface and detect its presence in the composite samples. XRD with specifications (maximum, output >3 KW, voltage > 60 kV, current > 60 mA) was used to analyze the composite sample (i.e., control without added *f*-CNT and best sample *f*-MWCNTs added). XRD analysis showed a noticeable difference for *f-MWCNTs* presence and crystalline nature of the composite, as shown in Fig 5. The existence of XRD peaks for 0.4 wt.% composite sample (Fig 5b) as compared to the control virgin sample (Fig 5a) demonstrate different surface structures, as can be witnessed. The higher peaks of 0.4 wt. % *f-MWCNT* sample around 23 (**2θ)** manifest the presence of *f-MWCNT* and its distribution of nanoparticles on the glass fiber substrate [33, 45]. The significant difference between the two curves indicates the presence of *f-MWCNTs* in one sample. The sample modified with *f-MWCNTs* showed better crystallinity than those with no *f-MWCNTs* added. The proper distribution of *f-MWCNTs* on the surface sample increases its crystallinity and strength [33].

### 3.4 Tensile strength

**Fig 6:**
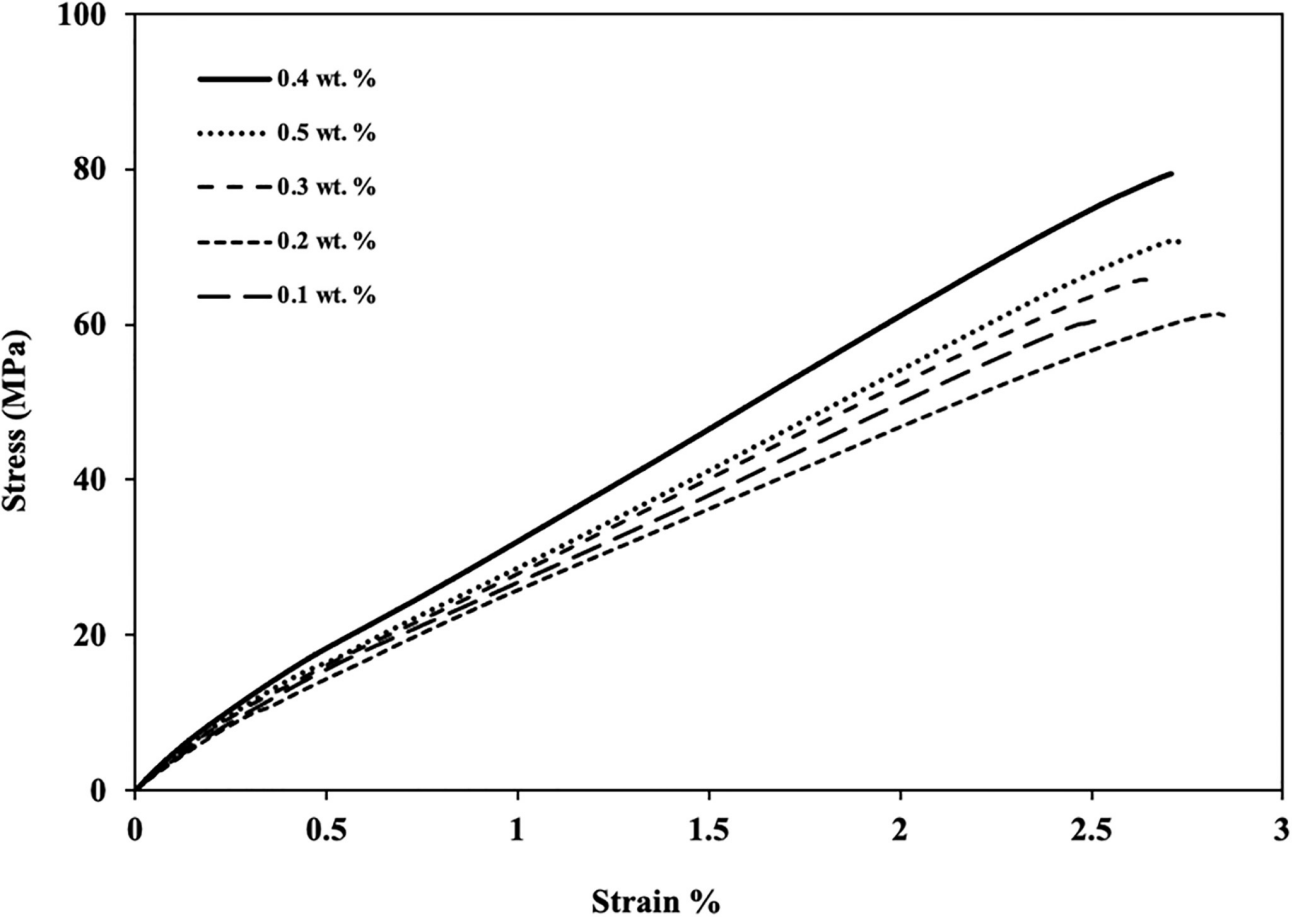
Stress-strain curves of different wt. % *f-MWCNTs* based modified GFRPs.

**Fig 7:**
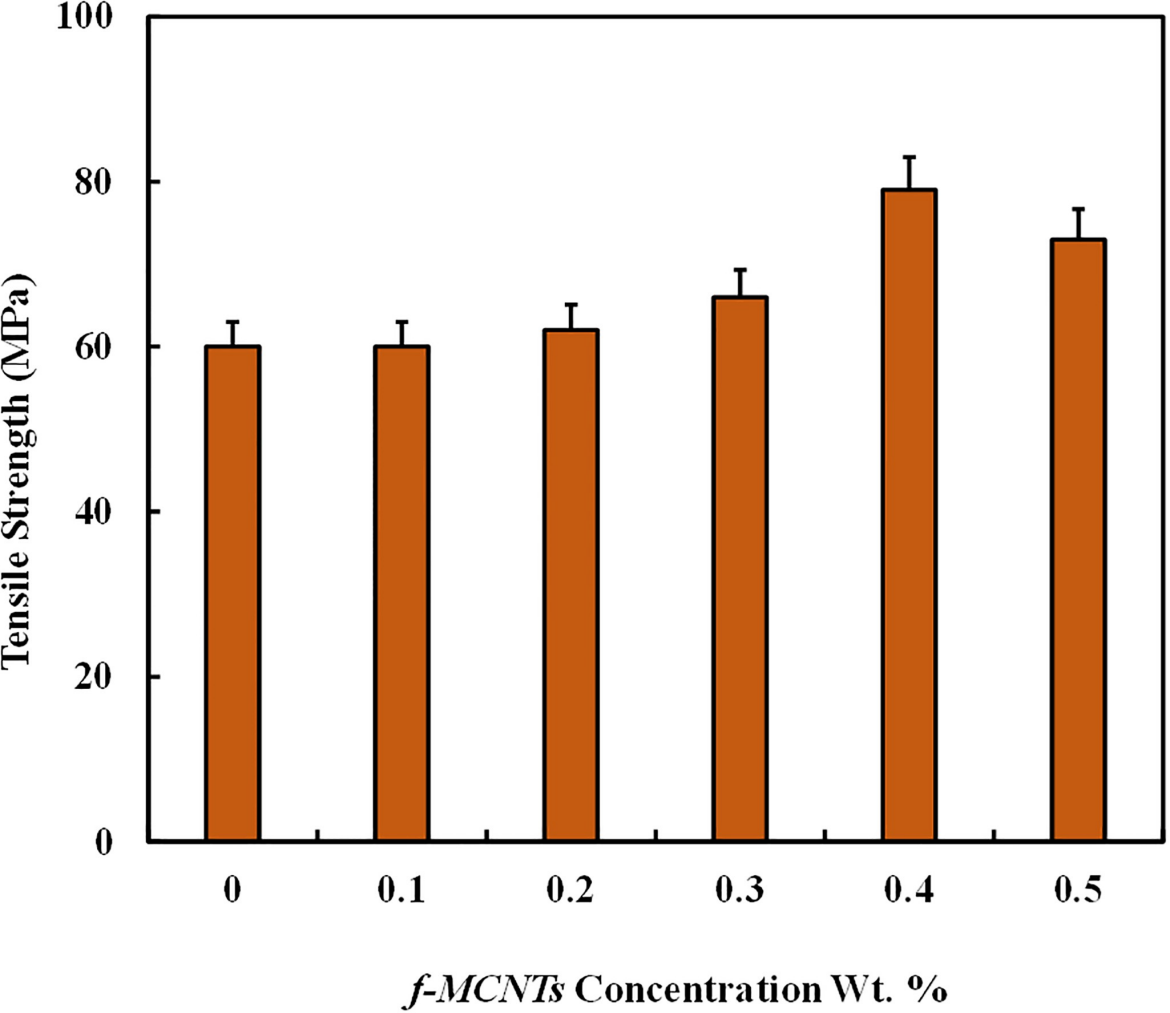
Effect of *f -MWCNTs* on tensile strength of GFRPS.

Tensile tests were conducted according to ASTM D3039 on a universal testing machine. Load-displacement curves and tensile strength for different concentrations of *f-MWCNTs* were recorded. The specimen was rectangular sheets of dimension 250×20×2.5 mm^3^, as shown in Fig 2b, and tested at a 2 mm/min loading speed. Fig 6 shows the stress-strain behavior, while Fig 7 shows the ultimate tensile strength of *f-MWCNTs* modified GFRPs.

Fig 6 shows that incorporating small concentrations of *f-MWCNTs* in polyester resin as nano-reinforcement results in forming *f-MWCNTs* modified GFRPs with improved mechanical properties. Analysis of the tensile strength of different composites with varying *f-MWCNTs* wt. % (Fig 7) showed the composite with 0.4 wt.% *f-MWCNTs* exhibited the highest tensile strength of 79 MPa. Furthermore, the sample with 0 and 0.1 wt. % have nearly same strength because the concentration of nanoparticles is so tiny that its effect is negligible in 0.1 wt % sample. Moreover, higher weight fractions of *f-MWCNTs* are not used because these nanoparticles agglomerate due to high attractive forces between them. The slope of the curves, which represents the stiffness, is highest for the composite with a 0.5 wt. % composite due to the brittle nature of *f-MWCNTs*. Comparing the virgin composite having 0 wt. % *f-MWCNTs* with composite having 0.4 wt. % *f-MWCNTs* improvement of 31.66% in the tensile strength was achieved from 30 to 79 MPa. Fig 7 also shows that increasing the concentration of *f-MWCNTs* from 0 to 0.4 wt. % the tensile strength of composites increases because *f-MWCNTs* increase the interaction between fibers and resin but on 0.5 wt. %, the strength decreases due to agglomeration of *f-MWCNTs* and voids formation, as shown in Fig 3. As the concentration of nanoparticles increases, their interaction with the polyester media weakens, and the attractive forces among nanoparticles overcome the effect of functionalization.

Wang *et al*. [12] showed that a similar trend was obtained with MWCNTs/fiber cloth/vinyl resin composite prepared by vacuum-assisted resin infusion method, and the composite with 0.3 wt. % MWCNTs showed the highest tensile and flexural strength. Ramlee *et al*. [15], from his work on Epoxy/Glass Fiber/ CNTs composite, showed that the tensile strength of the composites increased up to CNTs loading of 1 vol. %, and decreased at 1.5 vol. %. Improved tensile strength of GFRPs is due to the high modulus of *f-MWCNTs* and high surface area, which causes the interfacial bonding between the resin and fibers to be stronger. Various reasons could be possible for the decreased tensile strength at a high loading of *f-MWCNTs* which mainly depends on the type of resin, fiber reinforcement, types of CNTs used, and preparation method of composites. Agglomeration of CNTs in the resin matrix, improper wetting, and voids formation in the composite could be the main reason for the decreased tensile strength. Data graphed in Fig 7 conclude that the tensile strength of the developed polymeric composite material increases with an increase in the concentration of *f-MWCNTs*, but the type of material used in combination, also plays an important role.

### 3.5 Impact energy test

**Fig 8:**
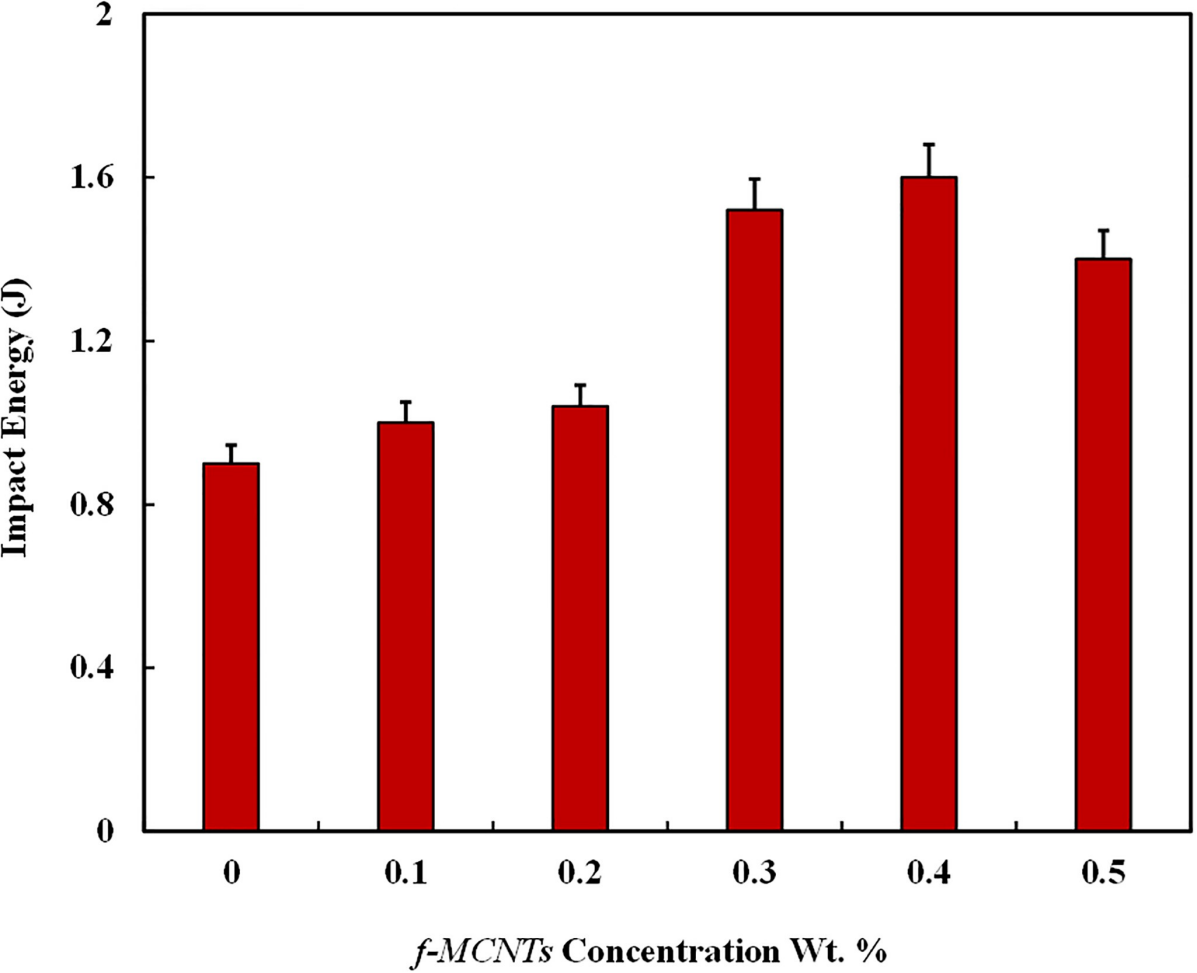
Impact strength of *f-MWCNTs* modified GFRPs.

Charpy Impact test was conducted according to ASTM D-256 on Charpy impact tester with sample dimension of 48 × 8 × 2.6 mm^3^ as shown in Fig 2c. Tests were performed on the pendulum impact tester PIT Series, U-shaped pendulum of up to 150 J, and results were obtained in the form of energy absorbed by samples during fracture. Fig 8 shows the effect of *f-MWCNTs* concentration on the impact strength of the composites as the impact strength is the measure of toughness or the energy absorbed by the material before it breaks with the load. Composite with 0.4 wt. % showed the highest impact energy of 1.6 Nm with 77% improvement compared to the composites with 0 wt. %. *f-MWCNT*. Similarly, the impact strength at 0.5 wt. % is decreased by 13%.

The impact energy of the composites increases up to 0.4 wt. % of *f-MWCNTs* loading because of exceptional properties of CNTs such as high surface area, making the strong interfacial bonding between resin and fibers and high flexibility. On the other hand, as the concentration of nanoparticles was increased its interaction with the polyester media was weakened and the effect of functionalization was overcome by the attractive forces among nanoparticles. Hence, the agglomeration of nanoparticles and void formation in the sample of 0.5 wt. % the impact energy is decreased.

### 3.6 Thermogravimetric analysis

**Fig 9:**
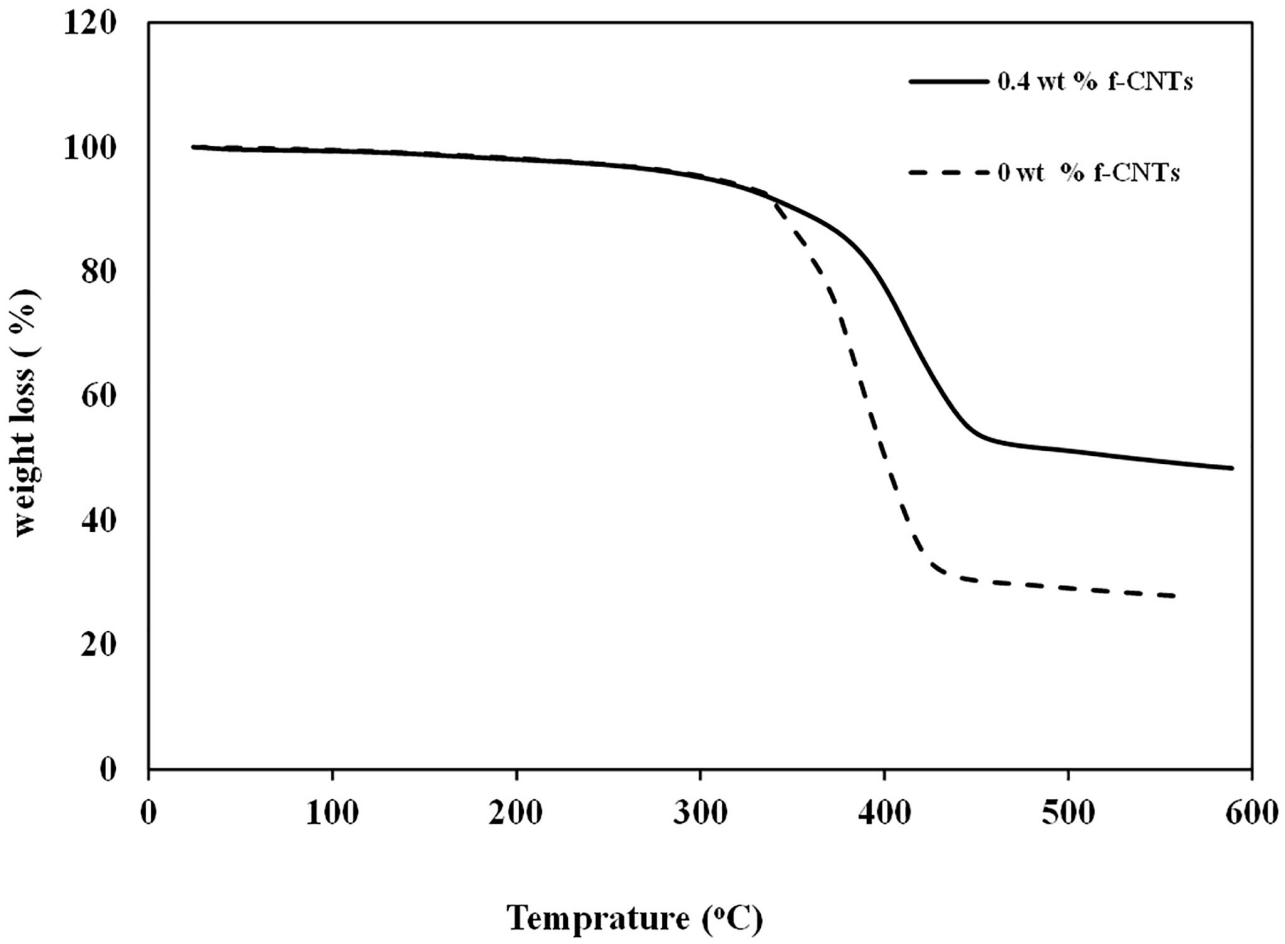
TGA for pure GFRPs and *f-MWCNTs* modified GFRPs.

Thermogravimetric gravity analysis (TGA) in the presence of N_2_ gas was done to assess the degradation behavior of the developed composite samples for further applications. The decomposition behavior and thermal stability of the selected samples (0 wt. % *f-MWCNTs* and 0.4 wt.% *f-MWCNTs*) were tested from room temperature to 600°C at a heating rate of 10°C/min under nitrogen environment. The thermal stability of the composite was measured by heating it at a constant rate and monitoring the change in weight simultaneously. Fig 9 evaluated TGA analysis under nitrogen to assess the degradation behavior of composite samples. The dashed line in Fig 9 shows the weight loss versus temperature curve for the GFRP having no *f-MWCNTs*. It is visible that for the 5% weight loss, the temperature is 300 °C, and maximum degradation occurs at 360 °C.

The solid line in Fig 9 shows the weight loss versus temperature curve for the GFRP having 0.4 wt. % *f-MWCNTs*, and from the curve, it is visible that for the 5% weight loss, the temperature is 305 °C, and maximum degradation occurs at 390 °C. These TGA results verified that the thermal stability of the sample containing functionalized CNTs is higher than that of no *f-MWCNTs* added and thus degraded at a lower temperature.

By comparing the obtained results with *f-MWCNTs*, the modified GFRPs and analyzing the effect of the different integrated bicomponent on the tensile strength and stability (i.e. resistance to temperature degradation), to those reported in the literature, as shown in Table 2, our values are comparatively higher.

**Table 2:** Comparison of the obtained tensile strength and TGA results by the application of different binary fillers material.

Materials	Composition of CNTs	Tensile Strength MPa	TGA Degradation °C	Ref.
Isotactic polypropylene	2.5% MWCNTS	31.5	340	[33]
Polyarylene ether nitriles	3% MWCNTS	70	446	[32]
Polyoxytetramethyleneglycol	2% MWCNTS	40	419	[34]
Polyvinyl chloride	0.25% SWCNTS	21	301	[31]
EPON 862 + EPI-W	1%MWCNTS	90	-	[18]
Glass fiber/vinyl ester composite	0.015%SWCNTS	30	-	[46]
Cement, silicon fume, quartz sand, silica sand, superplasticizer	0.01MWCNTS	11.1	-	[47]
***Polyester resin***	***0*.*4% MWCNTS***	***79***	***360***	***This work***

Results presented in Table 2 were compared with some of the relevant work that used different types of material frameworks to intake *f-MWCNTs* as filler to enhance the mechanical properties and the effect of temperature on them. All of the work mentioned above gives us a variety of materials with different concentrations of carbon nanotubes. The tensile strength increases with increasing concentration of MWCNTs, but the type of material also plays its role. According to the direct relation of MWCNTs concentration and the strength of the composite the best result has been shown by the EPON 862 + EPI-W based composite which showed a strength value of 90 MPa with 1% MWCNTs compared to our work which showed a relatively closer strength value with 0.4% concentration values. This result is, of course, greatly affected by the type of material used in the composite to be micro-filled by the MWCNTs, but degradation effects at higher temperature still need to be evaluated. In Table 2, the tensile strength of the Polyoxytetramethylene glycol-based fiber is 40 MPa has 2% of CNTs but if the work of Bikiaris *et al*. [33] is observed, where the MWCNTs concentration is greater than the former i.e., 2.5%, the values of the tensile strength seems to be less in comparison. This can be because of the material being used, isotactic polypropylene, affected the mechanical strength of the composite in some way. The temperature effects and the degradation points are also mentioned, as they are also very much concerned with the type of material and the concentration of MWCNTs. Compared to the work mentioned above, our degradation value is 360 °C, at which the degradation of the polymer composite happens. Compared to tabulated values, current work has demonstrated relative enhanced values by using a very simpler, less costly, and affordable method.

### 3.7 Multiple layer deposition analysis

Although during the development of the polymeric composite material, 0.4 wt % *f-MWCNTs* was found to be the best in terms of their mechanical properties, the extent of secondary components i.e., glass fiber, was also needed to be investigated. A compressive strength test for multilayer deposition was performed according to ASTM D695 with cylindrical sample dimensions of 12.7 mm in diameter and 25.4 mm long, as shown in Fig 2d. The specimen is placed between compressive plates of a universal testing machine parallel to the surface. The specimen is then compressed at a uniform rate.

**Fig 10:**
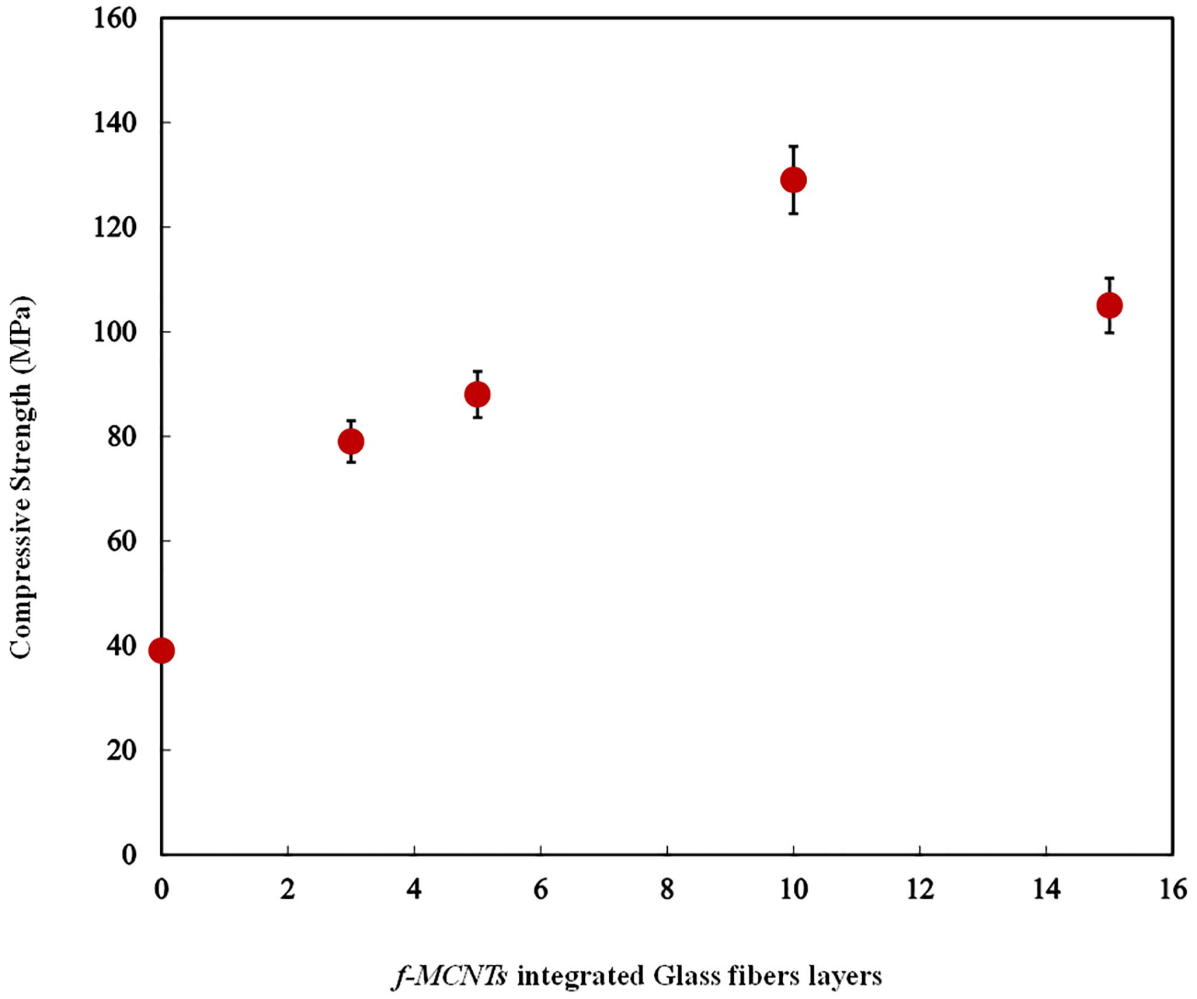
Compressive strength as a function of glass fiber layers modified GFRPs.

In this regard, effect of the additional layers of the glass fiber in the composite containing 0.4 wt. % *f-MWCNTs* was investigated and its effect on the compressive strength of the composite was measured as shown in Fig 10. It is evident from the data presented in Fig 10 that increasing layers of glass fibers up to 10 resulted in high compressive strength. However, as the number of glass fiber layers increased beyond then, the compressive strength of the composite material got reduced. The possible reason behind this failure beyond a certain number of layer addition is that a greater number of layers were unable to stick together and eventually cracked and broke into pieces at high impact load when applied [19, 48].

Conclusively, in the current research work, various tests were done to confirm the presence of *f-MWCNTs* in GFRPs, which was added to improve the mechanical properties of the developed composite. The strength of the composite was tested by tensile test, which showed excellent results of 79 Mpa. The polyester resin reinforced with fibers has quite promising mechanical abilities, but the infusion of *f-MWCNT* as a micro filler further enhanced the tensile strength, impact bearing strength, and resistance to degradation at a higher temperature. The concentration of *f-MWCNT* was varied to get the best results.

## 4. Conclusions

The current work investigated the development and properties of hybrid glass fiber reinforced polyester resin (GFRPs) composite. The composite integrated functionalized carbon nanotubes (*f-MWCNTs*) with glass fiber (GF) using polyester resin as a media and hand lay-up method in the form of rectangular sheets. It was concluded that with the addition of *f*-CNT, the tensile properties of GFRPs were significantly increased. The tensile strength was increased by 31.66% when the concentration was increased from 0.1 wt.% to 0.4 wt.%. Further increase in the concentration to 0.5 wt.% decreased the tensile strength by 10% due to agglomeration of *f-MWCNTs*. Similarly, with the infusion of 0.1 wt.% *f-MWCNTs* in GFRPs, the impact energy initially increased by 11.11% from 0.9 to 1.04 J and at 0.4 wt.% it was significantly increased by 77% compared to control sample. However, further increase to 0.5 wt.% decreased the impact energy by 12.5%. These results showed that *f-MWCNTs* deposited on the fiber glass surface effectively expanded interface area resulting in strong interfacial bonding; thus, resulted an increased in tensile strength and impact energy. The decreased in these properties was due to the agglomeration of fillers and formation of voids in developed composite due to the high viscosity of the *f-MWCNTs* and resin mixture. Furthermore, TGA of 0.4 wt. % *f-MWCNT* also showed the thermal stability till 390 °C as measured through thermogravimetric analysis (TGA) which confirmed that modified surface of the developed composite degraded less compared to the control surface. Deposition of extra 10 layers initially increased the composite strength from 40 MPa to 128 MPa, however further increase in layers to 15 resulted in decrease in strength to 100 MPa due to the poor interaction between the resin and glass fibers.

## Supporting information

S1 File (XLSX)

S2 File (XLSX)
